# Fungal Spore Richness and Abundance of Allergenic Taxa: Comparing a Portable Impactor and Passive Trap Indoors and Outdoors in an Urban Setting

**DOI:** 10.1007/s00248-024-02358-3

**Published:** 2024-02-23

**Authors:** Nicholas T. Minahan, Chi-Hsien Chen, Yu-Chen Chuang, Kun-Hsien Tsai, Wei-Chiang Shen, Yue Leon Guo

**Affiliations:** 1 Institute of Environmental and Occupational Health Sciences, National Taiwan University, Taipei, Taiwan; 2 Present Address: National Institute of Environmental Health Sciences, National Health Research Institutes, Miaoli, Taiwan; 3 Department of Environmental and Occupational Medicine, National Taiwan University (NTU) College of Medicine and NTU Hospital, Taipei, Taiwan; 4 Department of Plant Pathology and Microbiology, National Taiwan University, Taipei, Taiwan; 5 National Institute of Environmental Health Sciences, National Health Research Institutes, Miaoli, Taiwan

**Keywords:** Airborne fungi, Amplicon sequencing, Bioaerosol sampling, Environmental DNA, Rarefaction

## Abstract

Fungal spores are common airborne allergens, and fungal richness has been implicated in allergic disease. Amplicon sequencing of environmental DNA from air samples is a promising method to estimate fungal spore richness with semi-quantification of hundreds of taxa and can be combined with quantitative PCR to derive abundance estimates. However, it remains unclear how the choice of air sampling method influences these estimates. This study compared active sampling with a portable impactor and passive sampling with a passive trap over different durations to estimate fungal spore richness and the abundance of allergenic taxa. Air sampling was conducted indoors and outdoors at 12 residences, including repeated measurements with a portable impactor and passive traps with 1-day and 7-day durations. ITS2 amplicon sequence data were transformed to spore equivalents estimated by quantitative PCR, repeated active samples were combined, and abundance-based rarefaction was performed to standardize sample coverage for estimation of genus-level richness and spore abundance. Rarefied fungal richness was similar between methods indoors but higher for passive traps with a 7-day duration outdoors. Rarefied abundance of allergenic genera was similar between methods but some genera had lower abundance for passive traps with a 1-day duration, which differed indoors and outdoors indicating stochasticity in the collection of spores on collocated samplers. This study found that similar estimates of fungal spore richness and abundance of allergenic taxa can be obtained using a portable impactor or a passive trap within one day and that increased passive sample duration provides limited additional information.

**Supplementary Information:**

The online version contains supplementary material available at 10.1007/s00248-024-02358-3.

## Introduction

Fungal spores are common airborne allergens [1–4] and represent complex mixtures that require high-throughput methods for their characterization. To this end, environmental DNA (eDNA) amplicon sequencing enables semi-quantification of up to hundreds of fungal taxa in low-biomass air samples despite limitations in species-level identification [5]. Taxonomic richness is a facet of biodiversity that has been hypothesized to play a role in the etiology of allergic disease [6], and indoor fungal richness has been implicated in allergic asthma [7–9]. Perhaps more important is the abundance of airborne spores from allergenic fungi, particularly *Alternaria*, *Cladosporium*, *Penicillium*, and *Aspergillus* spp., which have consistently been linked to asthma and other atopic diseases [1–4]. A related challenge for eDNA analysis of airborne fungal spores has been quantification of diverse taxa, for which universal quantitative PCR (qPCR) has been combined with amplicon sequencing to estimate the abundance of individual taxa [10].

Various types of active [11–19] and passive [11, 16, 17, 20–24] air sampling devices have been used to collect airborne fungal spores for eDNA analysis, but critically, vary in the amount of sample effort both in terms of the abundance of fungal spores collected and sample duration. Sample coverage, which is considered an objective measure of sample completeness [25], has been overlooked. Low-cost passive spore traps based on dry deposition are particularly appealing for large-scale epidemiological studies to measure individual-level exposure to airborne fungal spores indoors and may also be used outdoors. However, congruence in the estimation of fungal spore richness and abundance of individual taxa with active measurements taken by portable air sampling devices such as portable impactors remains unclear. It is also unclear whether long sample durations previously used with passive traps are necessary to provide complete estimates of fungal spore richness. This study aimed to compare a portable impactor and passive trap over different durations to estimate fungal spore richness and abundance of allergenic taxa indoors and outdoors.

## Methods

### Air Sampling and DNA Extraction

**Fig. 1:**
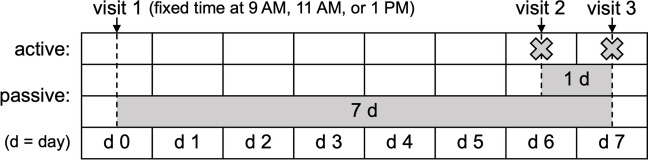
Air sampling scheme per three residences, performed indoors and outdoors over 1 week. Passive traps were deployed on day 0 and day 6 and collected on day 7. Active samples taken with a portable impactor on day 6 and day 7 were combined for comparison with passive traps

Air sampling was conducted at 12 residences in Taipei City and New Taipei City in northern Taiwan during August and September 2020 (Supplementary Fig. 1). Sampling was conducted in three residences at a time, with each residence visited three times (days 0, 6, and 7) at a fixed time at 9 AM, 11 AM, or 1 PM (Fig. 1). Air sampling was performed indoors in the living room and outdoors on the balcony or at ground level.

Each sampling method utilized sterile Vaseline-coated Petri dishes (VCPDs), which were prepared under laminar flow by evenly applying a thin layer of Vaseline (Sigma-Aldrich, USA, product # 16415) to 90 × 15 mm Petri dishes (Alpha Plus, Taiwan) using a sterile cotton swab. VCPDs were sealed using Parafilm M (Amcor, USA) and stored inside resealable plastic bags.

During each visit, active sampling was performed simultaneously indoors and outdoors using MicroBio MB2 Bioaerosol Sampler (Cantium Scientific, UK) devices with 400-hole heads, which were vertically positioned at 1.5 m and used to sample 1000 L of air (100 L/min) on VCPDs (Supplementary Fig. 2). Sample heads were sanitized using 75% ethanol before each sample. Occupants and personnel were not in the room during sampling (with a 1-min delayed start), and fans were turned off. After sampling, VCPDs were recapped and sealed using Parafilm M and transported at ambient temperature to the laboratory for immediate DNA extraction each day.

Passive sampling was also performed indoors and outdoors using VCPDs as passive traps. During the first visit, sampling apparatuses were installed in each location with a data logger (HOBO UX100-003, Onset, USA) to record temperature and relative humidity at 5-min intervals. Indoors, an area near a wall was selected away from doors, windows, and avoiding fans, and two sampling apparatuses were suspended from the ceiling by adhesive hooks, placed approximately 10 cm from the wall and apart from each other, with VCPDs positioned 1.5 m from the floor. Outdoor sampling apparatuses were secured to clothes hangers and hung out of direct sunlight (Supplementary Fig. 2). VCPDs were deployed on day 0 and day 6 and collected on day 7 for 7-day and 1-day sample durations, respectively (Fig. 1). Occupants were asked to avoid walking near and directing fans towards the passive traps.

DNA extraction was performed using the DNeasy PowerSoil Pro Kit (Qiagen, Germany), following the manufacturer’s instructions. Vaseline was collected from plates under laminar flow using a sterile cotton swab, with two complete passes (one for each side of the swab); after each pass, Vaseline-coated cotton was cut using ethanol-cleaned scissors onto the VCPD and transferred into a PowerBead Pro Tube containing Solution CD1 using ethanol-cleaned forceps. Lysis was performed using a Mini-Beadbeater-16 (BioSpec, USA) for 2 min. DNA was eluted using 50 µL of the kit’s elution buffer and stored at − 20 °C. Unexposed VCPDs were also extracted weekly to monitor for contamination.

### Quantitative PCR

Quantitative PCR was performed to estimate the number of fungal spore equivalents referencing a standard constructed with genomic DNA extracted from a known quantity of conidia from a pure culture of *Cladosporium tenuissimum* (BCRC # 30812) cultivated on potato dextrose agarose (Neogen, USA) in the dark at 25 °C. Fresh conidia were harvested by flooding plates with sterile phosphate-buffered saline containing 0.05% Tween 20 (Sigma-Aldrich), filtered through several layers of sterile Miracloth (Millipore, USA), and adjusted to a concentration of 5.0 × 10^6^ conidia/mL using a hemocytometer. Then, 1 mL of conidia were pelleted at 15,000 × *g* for 5 min, the supernatant was removed, and a sterile cotton swab with its tip coated with Vaseline from an unexposed VCPD was used to recover conidia for DNA extraction as before, except DNA was eluted using 100 µL of elution buffer for a final concentration of 5.0 × 10^4^ spore equivalents/µL. Duplicate extractions were performed and pooled 1:1 (vol/vol) and diluted to generate standards. SYBR Green-based qPCR was performed targeting the fungal internal transcribed spacer region 2 (ITS2) with the ITS86F/ITS4 primer pair [26, 27], which was also used for PCR. Triplicate 10 µL reactions were prepared with 1X iTaq Universal SYBR Green Supermix (Bio-Rad, USA), 0.3 µM of each primer, PCR-grade water, and 2 µL of DNA template. Each assay included six standards from 10^5^ to 10^0^ spore equivalents and no template controls. Quantitative PCR was performed using the Bio-Rad CFX384 system with 95 °C for 5 min, 40 cycles of 95 °C for 5 s and 58 °C for 60 s, followed by a melt curve from 65 to 95 °C with increments of 0.5 °C for 5 s. Estimation of spore equivalents was performed using Bio-Rad CFX Maestro software. For samples with C_T_ values with a standard deviation (SD) ≥ 0.2, one value was excluded to yield the lowest SD, and mean spore equivalents were used to back-calculate total spore equivalents.

### Amplicon Sequencing

Duplicate PCR reactions (25 µL) were performed for each sample, consisting of 1X NEBNext Ultra II Q5 Master Mix (New England Biolabs, USA), 0.5 µM of each primer (ITS86F/ITS4) with Illumina adapter overhangs (Integrated DNA Technologies, USA), and 10 µL of DNA template. PCR was performed in a Biometra TRIO thermal cycler (Analytik Jena, Germany) with 98 °C for 30 s and 30 cycles of 98 °C for 10 s, 50 °C for 30 s, 72 °C for 15 s, and final extension at 72 °C for 5 min. Each round of PCR included a positive control with 10^4^ spore equivalents and a no template control. PCR amplification was confirmed using agarose gel electrophoresis and SYBR Green staining. Duplicate reactions were pooled and purified using AMPure XP beads (Beckman Coulter, USA) with a 0.9 bead-to-sample ratio. The amount of DNA in purified PCR products was quantified using the Epoch Spectrophotometer System (BioTek Instruments, USA). Index PCR reactions (50 µL) consisted of 1X KAPA HiFi HotStart ReadyMix (Roche, Switzerland), 0.5 µM of Index 1 (i7) and Index 2 (i5) primers from Nextera XT Index Kit v2 (Illumina, USA), PCR-grade water, and 100 ng of purified PCR product. Index PCR was performed using a T1 thermocycler (Biometra, Germany) with 95 °C for 3 min and 6 cycles of 98 °C for 20 s, 55 °C for 30 s, 72 °C for 30 s, and final extension at 72 °C for 5 min. Indexed products were purified with AMPure XP beads and quantified with the Qubit dsDNA HS Assay kit (Invitrogen, USA) using a Qubit 2.0 Fluorometer (ThermoFisher Scientific, USA). Purified libraries were then pooled at equimolar amounts and assessed using the 2100 BioAnalyzer (Agilent, USA), Qubit, and qPCR with the KAPA Library Quantification Kit (Roche). Sequencing was performed using the Illumina MiSeq platform with the MiSeq Reagent Kit v3 (600-cycle) at the Technology Commons (College of Life Science, National Taiwan University) following the manufacturer’s instructions with a 20% PhiX spike-in.

### Sequence Data Processing and Statistical Analysis

Demultiplexed sequence data, with adapter sequences and PhiX removed using Illumina MiSeq Reporter software, were processed using dada2 1.28.0 [28] in R 4.3.0 (https://www.R-project.org/). Reads containing ambiguous bases were removed, and cutadapt 4.0 [29] with Python 3.9.12 (https://www.python.org/) was used to remove ITS86F and ITS4 primer sequences from all reads. Forward and reserve reads with more than two expected errors were removed. All bases were used to learn the error rates, sample inference with the dada function was performed with pseudo-pooling, forward and reverse reads were merged with at least 100 bases of overlap, and chimeras were removed. Taxonomy was assigned to amplicon sequence variants (ASVs) referencing the UNITE general FASTA release for eukaryotes 2 [30] version 29.11.2022 with default bootstrap confidence and reverse-complement sequences considered.

Resulting datasets were imported to phyloseq 1.44.0 [31] for further processing. ASVs with kingdom-level assignments other than Fungi were removed, ASVs with identical genus-level assignments were combined while discarding ASVs with missing assignments, and counts were transformed to relative abundance. At first, qPCR-estimated spore equivalents for each sample were multiplied by relative abundance values to estimate spore equivalents for each taxon [10], but this resulted in decimal values that are not suitable for rarefaction-based methods. Rounding to the nearest integer reduces richness in samples with low abundance (due to rare taxa with values ≤ 0.5 being rounded to 0). Rounding up preserves rare taxa as singletons but increases abundance; therefore, an adjusted abundance (${A}_{adj}$) was calculated that subtracts this rounding error from the original abundance ($A$) as follows: for a given sample, ${A}_{adj}=A-\left(\left({\sum }_{i=1}^{S}\lceil{R}_{i}\times A\rceil\right)-A\right)$ where ${R}_{i}$= relative abundance of each taxon and $S$ = observed richness. Here, abundance data are in units of spore equivalents, and adjusted abundance values were used to estimate spore equivalents for each genus in a given sample by $\lceil{R}_{i}\times {A}_{adj}\rceil$. Repeated active samples on day 6 and day 7 were combined for comparison with passive samples.

Sample coverage was computed using iNEXT 3.0.0 [32] with abundance data. Samples with coverage less than 97.5% were excluded from further analysis, as were paired samples from the same residences. Rarefaction was used to estimate richness (*q* = 0) at a coverage of 97.5%. Subsample sizes ($m$) corresponding to a coverage of 97.5% were then used to calculate rarefied spore equivalents for each genus in a given sample by ${R}_{i}\times m$ (values were not rounded). Pseudocounts of 1 were added to all values of genus-level abundance data before log10-transformation. Fungal genera with airborne allergens listed in the World Health Organization/International Union of Immunological Societies allergen nomenclature database [33] that were present in at least half of all samples were examined.

Data visualization was performed with ggpubr 0.6.0 [34], and rarefaction curves were generated using ampvis2 2.7.32 [35]. Paired samples were compared using paired *t*-test for normal and homoscedastic data and Wilcoxon signed-rank exact test for non-normal and heteroscedastic data, and *p* values were adjusted for multiple comparisons using the Holm method (*p* values < 0.05 were considered significant). Comparisons between samples were stratified by sample location. Indoor/outdoor (I/O) ratios were also calculated for untransformed values of rarefied richness and abundance of allergenic genera. I/O ratios of rarefied abundance were compared between samples using Mann–Whitney *U* test, excluding data points with zero or undefined values.

## Results

Air sampling was completed at 12 residences, including ten apartments and two townhouses between 20 and 60 years old (Supplementary Table 1). At least one external window was reported to be kept open in each room sampled or in an adjoining room with open airflow, and indoor and outdoor temperatures remained similar throughout the sample period in each residence (Supplementary Table 1). Each room had a pedestal fan used in the daytime when occupied, and air conditioning units were absent or unused. Relative humidity was generally lower indoors than outdoors, except for residence no. 1, which had consistently higher indoor relative humidity (Supplementary Table 1) with other dampness-related indicators, including peeling plaster on the wall and a history of flooding. No visible mold was found in the areas where sampling was conducted.

Sequence data for 120 air samples produced 16.85 million raw paired reads (77,133 to 221,292 paired reads per sample) with 2.27 million paired reads (9297 to 36,849 paired reads per sample) after quality filtering. Following subsequent processing, a total of 2.01 million ASV counts were generated (7432 to 35,338 ASV counts per sample) for 21,284 ASVs (159 to 1235 observed ASVs per sample) with a mean length of 281 bp (100 to 461 bp). Among these, 19,905 ASVs had a kingdom-level assignment of Fungi (96.45% of total ASV counts), and 1544 genera were identified (90.47% of fungal ASV counts).

Individual active samples had a mean of 21,080 spore equivalents (296 to 339,932 spore equivalents) with 190 observed genera (70 to 348 observed genera) compared to 41,072 spore equivalents (747 to 115,533 spore equivalents) with 258 observed genera (199 to 313 observed genera) for 1-day passive samples and 115,392 spore equivalents (21,968 to 348,478 spore equivalents) with 299 observed genera (226 to 368 observed genera) for 7-day passive samples. Unexposed VCPDs had < 1 spore equivalent per µL of DNA. After estimation of genus spore equivalents with adjusted abundance, active samples had a median increase in abundance of 0.02% (interquartile range, IQR = 0.08%), which was 0.01% (IQR = 0.02%) for 1-day passive samples, and 0% (IQR = 0.01%) for 7-day passive samples. This compared to a median increase in abundance of 1.31% (IQR = 2.46%), 0.36% (IQR = 1.65%), and 0.15% (IQR = 0.27%) for active, 1-day passive, and 7-day passive samples, respectively, after estimation of genus spore equivalents with unadjusted abundance (Supplementary Fig. 3). Combined active samples had a mean of 50,112 spore equivalents (1925 to 436,537 spore equivalents) with a mean observed richness of 268 genera (134 to 436 observed genera) and a mean sample coverage of 99.17% (91.07 to 100%), which compared to 98.21% (72.17 to 100%) for 1-day passive samples and 100% for all 7-day passive samples (Supplementary Fig. 4). Two residences (no. 3 and no. 7) had samples with coverage < 97.5% and were excluded, and 60 samples from 10 residences were analyzed. Fungal spore abundance was similar between active and 1-day passive samples indoors and outdoors but higher for 7-day passive samples (Supplementary Fig. 5). Observed genus richness of fungal spores was similar between methods indoors and outdoors (Supplementary Fig. 6). 201 genera were identified in at least half of all samples. Eight allergenic genera were examined, including seven *Ascomycota* (*Alternaria*, *Aspergillus*, *Candida*, *Cladosporium*, *Curvularia*, *Fusarium*, and *Penicillium*) and one *Basidiomycota* (*Schizophyllum*). Differences were observed in the non-rarefied abundance of most allergenic genera between methods indoors or outdoors and were generally lowest for 1-day passive samples and highest for 7-day passive samples, except for *Schizophyllum* and *Penicillium*, which were consistently similar (Supplementary Fig. 7).

**Fig. 2:**
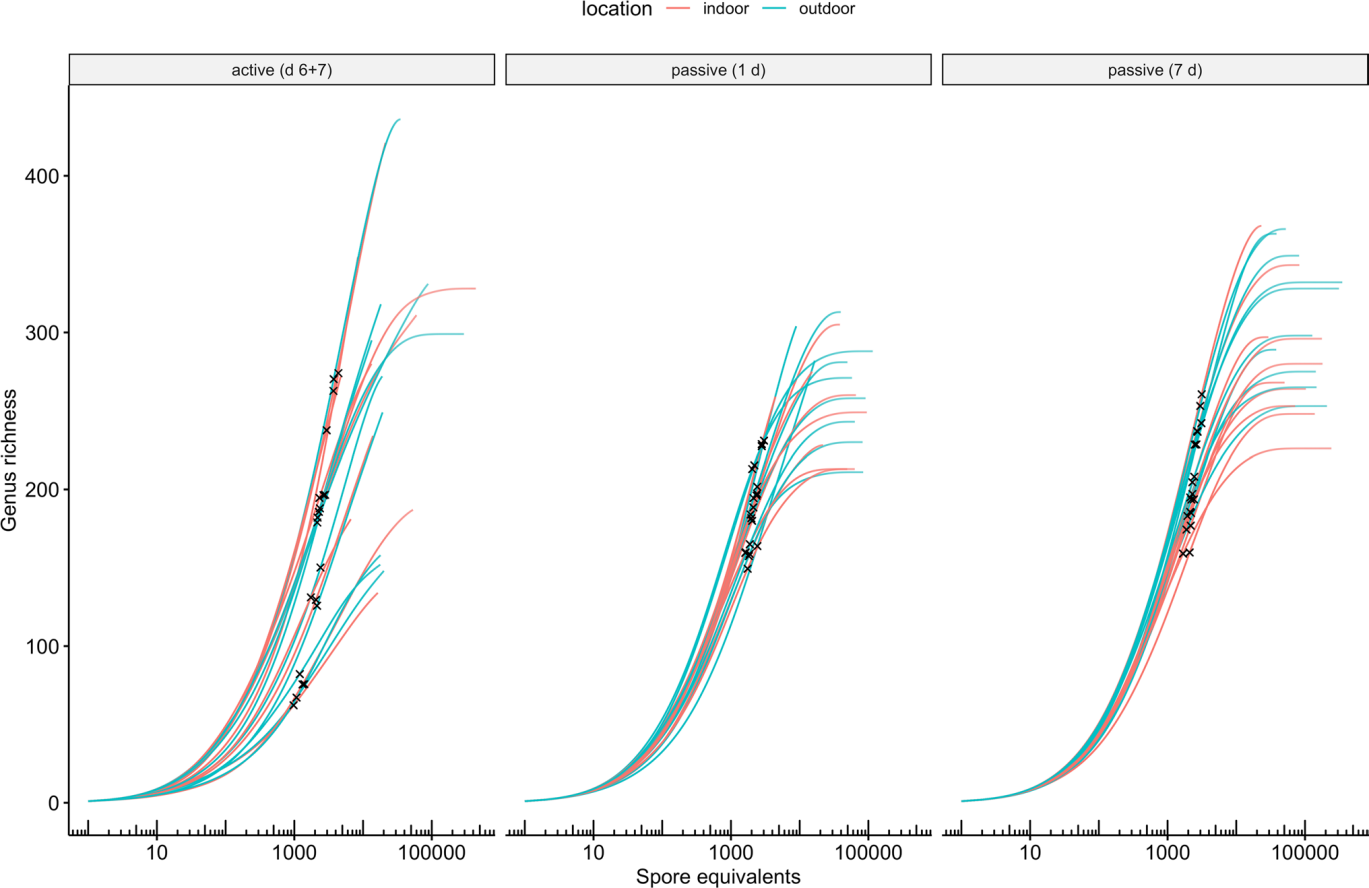
Rarefaction curves depicting the accumulation of fungal spore richness (genus richness) with increasing sample size (spore equivalents) for active (day 6 + 7) and passive (1-day and 7-day) air samples collected indoors and outdoors at 10 residences (*n* = 10 per group). Cross marks indicate subsample sizes corresponding to sample coverage of 97.5%

**Fig. 3:**
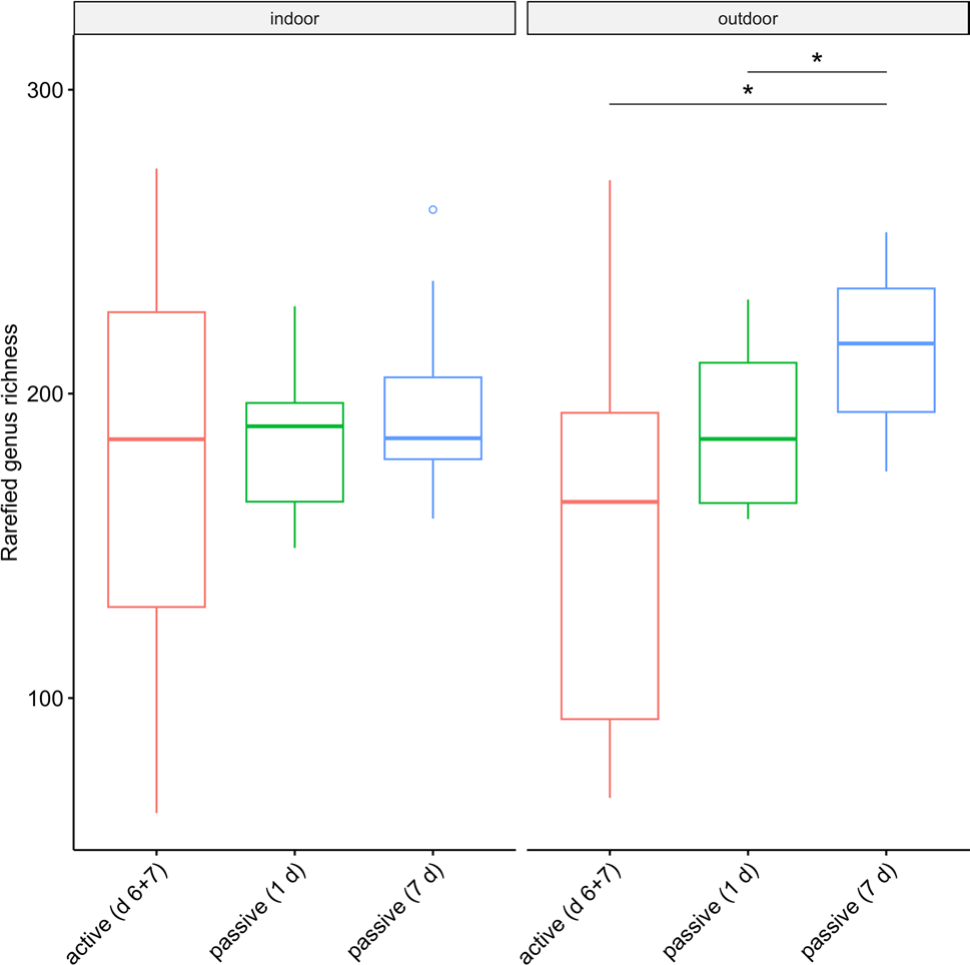
Rarefied genus richness of fungal spores between active (day 6 + 7) and passive (1-day and 7-day) air samples at a coverage of 97.5%, stratified by location (*n* = 10 per group). Horizontal lines indicate median, boxes indicate interquartile range (IQR), whiskers indicate values within 1.5 × IQR of Q1 and Q3, and open circles indicate outliers. Statistical comparisons were made using Wilcoxon signed-rank exact test, and *p* values were adjusted for multiple comparisons using the Holm method (*p* ≥ 0.05 not shown; *p < 0.05)

**Fig. 4:**
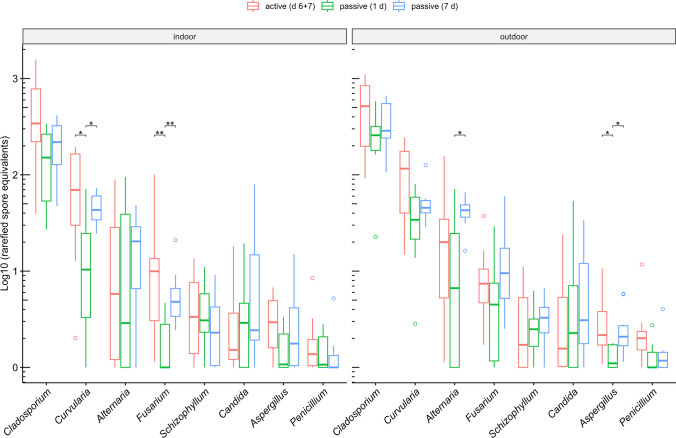
Rarefied abundance (log10-transformed spore equivalents) of allergenic fungal genera between active (day 6 + 7) and passive (1-day and 7-day) air samples at a coverage of 97.5%, stratified by location (*n* = 10 per group). Pseudocounts of 1 were added to all values before log10-transformation. Horizontal lines indicate median, boxes indicate interquartile range (IQR), whiskers indicate values within 1.5 × IQR of Q1 and Q3, and open circles indicate outliers. Statistical comparisons were made using Wilcoxon signed-rank exact test, and *p* values were adjusted for multiple comparisons using the Holm method (*p* ≥ 0.05 not shown; *p < 0.05; **p < 0.01)

A mean subsample size of 2280 spore equivalents (976 to 4378 spore equivalents) was used for rarefaction of samples to 97.5% coverage, which was similar between methods (Fig. 2; Supplementary Fig. 8). Rarefied richness was similar between methods indoors but was significantly higher for 7-day passive than 1-day passive and active samples outdoors (Fig. 3). I/O ratios of rarefied richness were higher for active than passive samples (Supplementary Fig. 9). Rarefied abundance of allergenic genera was similar between active and passive methods indoors and outdoors (Fig. 4). However, indoors, 1-day passive samples had a lower rarefied abundance of *Curvularia* and *Fusarium* than active and 7-day passive samples, and, outdoors, a lower rarefied abundance of *Alternaria* compared to 7-day passive samples and *Aspergillus* compared to active and 7-day passive samples (Fig. 4). I/O ratios of rarefied abundance were similar between methods for each allergenic genus, and only *Aspergillus* and *Schizophyllum* had median I/O ratios > 1 for each method (Supplementary Fig. 10).

## Discussion

This study found similar estimates of fungal spore richness between repeated active measurements with a portable impactor and passive traps with different sample durations indoors but higher richness with increased passive sample duration outdoors. To our knowledge, no prior study has directly examined fungal spore richness between active air samples and passive traps or the influence of passive sample duration on fungal spore richness. Considering the short sample duration with portable impactors in this study, it is remarkable that this method produced similar fungal spore richness estimates to passive traps and indicates that spore richness peaked during the sampling window in this study (approximately 9 AM to 1 PM). This is consistent with a study in Singapore that found airborne fungal richness to peak around solar noon [18]. Even so, it was necessary to combine repeated active measurements taken a day apart for estimates of richness comparable to 1-day passive traps. While this may indicate some daily variation in fungal spore richness, it may be limited, as 7-day passive traps did not reveal higher richness indoors. Further investigation is required to study changes in fungal spore richness at different temporal scales. In our previous study, fungal spore richness passively sampled in school classrooms was only found to differ at the phylum level between seasons [36].

Different types of passive traps have been used to sample fungal spores for eDNA analysis. Indoors, electrostatic dust collectors [8, 23, 36] and empty Petri dishes [20, 37–39] have been used with varying sample durations (e.g., 2 weeks [36], 3 weeks [39], 4 weeks [20, 37, 38], and 10 weeks [8, 23]). Remarkably, the present study found that a 1-day sample duration with a VCPD was sufficient to provide a complete estimation of fungal spore richness indoors. Outdoors, empty Petri dishes have also been used [20, 38, 39], as has a quartz fiber substrate with mineral oil for 4-week periods [17], Petri dishes and Whatman filters with and without different coatings in a field for 15 days [24], and a funnel trap with weekly collection [22]. Other types of spore traps are designed to collect rainfall (wet deposition) [16, 17, 21] but were not considered in this study. Higher richness observed for 7-day passive traps outdoors could partly be due to the increased collection of small insects that carry spores on their bodies (which were aseptically removed before DNA extraction if present) or debris. Higher richness for passive traps outdoors resulted in lower I/O ratios of richness compared to active samples. Detection of rare taxa collected earlier in the sample period is contingent on DNA preservation, which was found to be significantly reduced by day two for *Entomophaga maimaiga* conidia on a polypropylene surface exposed in a field [40]. UV light exposure and rainfall are among the factors influencing the preservation of fungal spores collected on passive traps outdoors, requiring an apparatus to shield dry deposition-based passive traps if they are to be used in the field.

Sample coverage was used as a benchmark to objectively compare samples of airborne fungal spores with large differences in abundance, which, to our knowledge, is a novel approach. First, sample coverage provides a measure to evaluate whether samples are of adequate size to provide a fair estimation of richness. Second, rarefaction (and extrapolation) can be performed post hoc to standardize sample coverage to where samples have the same proportional abundance of detected and undetected taxa [25]. At a coverage of 97.5%, detected taxa represent at least 97.5% of the abundance in a sample, and undetected taxa represent at most 2.5%. We performed rarefaction because extrapolating richness for low-coverage samples with more than double the reference sample is not reliable [32]. Also, extrapolation is based on the number of singletons and doubletons in a sample [25], but for samples with high abundance (where $A\gg S$), no singletons or doubletons are produced after transformation to spore equivalents, as observed for all 7-day passive samples. We performed rarefaction at a coverage that was relatively high across samples while excluding extreme outliers.

This study also found similarities between active and passive methods in the rarefied abundance of allergenic taxa. However, several taxa had lower abundance for 1-day passive traps. Despite different predominant collection mechanisms of impactors (inertial impaction) and passive traps (gravitational sedimentation/dry deposition), collection efficiencies similarly increase with spore size related to higher inertia [41] and settling velocities [17]. Wind turbulence additionally influences spore deposition on passive traps through inertial impaction [11] and displacement of settling spores. Passive traps with a 1-day collection period may be more affected by wind turbulence than passive traps with a 7-day collection period, which also collect spores during lag days with varying wind conditions. Other meteorological factors may also be related to temporal variation in spore abundance. For example, *Fusarium* spores are preferentially deposited by wet deposition [16], and rainfall could have reduced their abundance during the 1-day passive sample period (indeed, outdoor humidity was elevated at six residences during this period). Notably, allergenic taxa found to have decreased abundance with 1-day passive traps differed indoors (*Curvularia* and *Fusarium*) and outdoors (*Alternaria* and *Aspergillus*) and did not appear to be related to spore size, indicating stochasticity in the collection of spores on collocated samplers. *Alternaria* and *Curvularia* produce large multicellular conidia with aerodynamic diameters around 10 µm and high settling velocities (0.6 and 1.2 cm/s, respectively), whereas *Aspergillus* produce small unicellular conidia with an aerodynamic diameter around 5 µm and much lower settling velocity (< 0.1 cm/s) [17]. It is notable that *Penicillium*, with conidia similar to *Aspergillus*, had similar abundance between methods, as a gelatin filter-based passive trap was previously found to be deficient in its detection [11]. Higher indoor abundance of *Aspergillus* and *Schizophyllum* spores may indicate inadequate ventilation despite window opening. *Schizophyllum* basidiospores originate from outdoor sources (basidiocarps) and have a low settling velocity (< 0.01 cm/s) [17]; thus, like *Aspergillus* conidia, remain suspended in the air for longer periods. Others have reported increased abundance of allergenic genera in classrooms during occupancy due to particle resuspension [42]. *Aspergillus* spp. are ubiquitous saprotrophs that grow on building materials, but spore abundance for other common indoor molds, including *Cladosporium* and *Penicillium*, was not consistently high indoors. While visible mold was not found in these residences, it remains unclear to what extent visible surface mold contributes to fungal spore abundance indoors. A study conducted in residences around Taiwan found the surface density of culturable *Geotrichum* on walls positively correlated with airborne *Geotrichum* abundance, but this relationship was not observed for other culturable fungi [43]. Indoor fungal spore abundance can be reduced by improving ventilation, perhaps most effectively through the use of air-conditioning [44].

Various active devices have been used to sample fungal spores for eDNA analysis, including Hirst-type spore traps [14, 45], multi-stage impactors [12, 17, 42], high-volume electret filter air samplers [18, 46, 47], and cyclones [16, 48]. While some of these devices can be used indoors, their size, cost, and other factors make them less feasible for large-scale studies. Alternatively, others have similarly used a portable impactor with Petri dishes containing Vaseline [19, 44], and filter cassettes with sampling pumps have also been used indoors [13, 15]. Of note, a low-cost filter-based sampler operated with a conventional vacuum cleaner has been devised that may be used as an alternative to portable impactors with simple calibration [49]. The congruence between active devices in the estimation of fungal spore richness still requires investigation.

### Limitations

We were unable to visit residences every day for increased sampling frequency. We were also unable to conduct sampling at all residences concurrently, but an effort was made to complete this study in as short of a window as possible with fixed visiting times. Air-change rates, which vary with time in residences [50], were not measured to assess indoor ventilation.

We did not have biological replicates. For active samples, it is unclear whether combining samples collected simultaneously or sequentially would yield similar results to those collected a day apart, as combined bioinformatically in this study. To this point, it would be more economical to pool replicates upstream, for example, during DNA extraction [44].

Quantification of fungal spores is a major challenge with eDNA. This is in part due to the targeting of regions of ribosomal DNA (rDNA), with each organism varying in its number of rDNA tandem repeats [51], and is compounded by variation in cell number and ploidy. We followed others [10] in selecting a representative species (*C. tenuissimum*, a common air isolate) to produce a qPCR standard using DNA from a known quantity of conidia extracted in a similar manner to our samples. Importantly, this controlled for any potential decrease in DNA extraction efficiency (as compared to extraction of conidia alone) or PCR inhibition during quantification.

Additionally, this study did not consider spore morphology or congruence of amplicon sequencing with microscopic identification. The portable impactor used in this study does not allow for size selection and is not well suited for microscopic identification as it deposits spores over a large collection area (as opposed to a slit impactor). A few studies have compared amplicon sequence data to the microscopic identification of fungal spores in active air samples [14, 52, 53]. One study evaluated the same primer pair and sequencing platform used in this study and reported good concordance in the relative abundance of morphologically identifiable taxa between methods [14]. However, these studies consistently found that amplicon sequencing overestimated the relative abundance of large multicellular spores, including *Alternaria* and *Epicoccum* [14, 53], which needs to be addressed.

## Conclusions

In conclusion, this study found that similar estimates of fungal spore richness and abundance of allergenic taxa can be obtained using a portable impactor or a passive trap within 1 day and that increased passive sample duration provides limited additional information. These findings inform sampling methods for eDNA analysis of airborne fungal spores in different settings.

### Supplementary Information

Below is the link to the electronic supplementary material.

Supplementary file1 (PDF 8446 KB)

## Data Availability

Sequence data is available in the NCBI Sequence Read Archive under BioProject Accession Number PRJNA1026110.
