# Supplementary material for: Fungal Spore Richness and Abundance of Allergenic Taxa: Comparing a Portable Impactor and Passive Trap Indoors and Outdoors in an Urban Setting
Source: Microb Ecol. 2024 Feb 23;87(1):45. doi: 10.1007/s00248-024-02358-3 (PMC10891272; doi:10.1007/s00248-024-02358-3)
Supplement: Supplementary file 1 — Supplementary file1 (PDF 8446 KB) [file 248_2024_2358_MOESM1_ESM.pdf]

Supplementary Material

Fungal Spore Richness and Abundance of Allergenic Taxa: Comparing a Portable Impactor and Passive Trap Indoors and Outdoors in an Urban Setting

Nicholas T. Minahan[1,a], Chi-Hsien Chen[2], Yu-Chen Chuang[2], Kun-Hsien Tsai[1], Wei-Chiang Shen[3], Yue Leon Guo[1,2,4,*]

[1]Institute of Environmental and Occupational Health Sciences, National Taiwan University, Taipei, Taiwan

[2]Department of Environmental and Occupational Medicine, National Taiwan University (NTU) College of Medicine and NTU Hospital, Taipei, Taiwan

[3]Department of Plant Pathology and Microbiology, National Taiwan University, Taipei, Taiwan

[4]National Institute of Environmental Health Sciences, National Health Research Institutes, Miaoli, Taiwan

[a]Present address: National Institute of Environmental Health Sciences, National Health Research Institutes, Miaoli, Taiwan

[*]Correspondence to Yue Leon Guo, leonguo@ntu.edu.tw

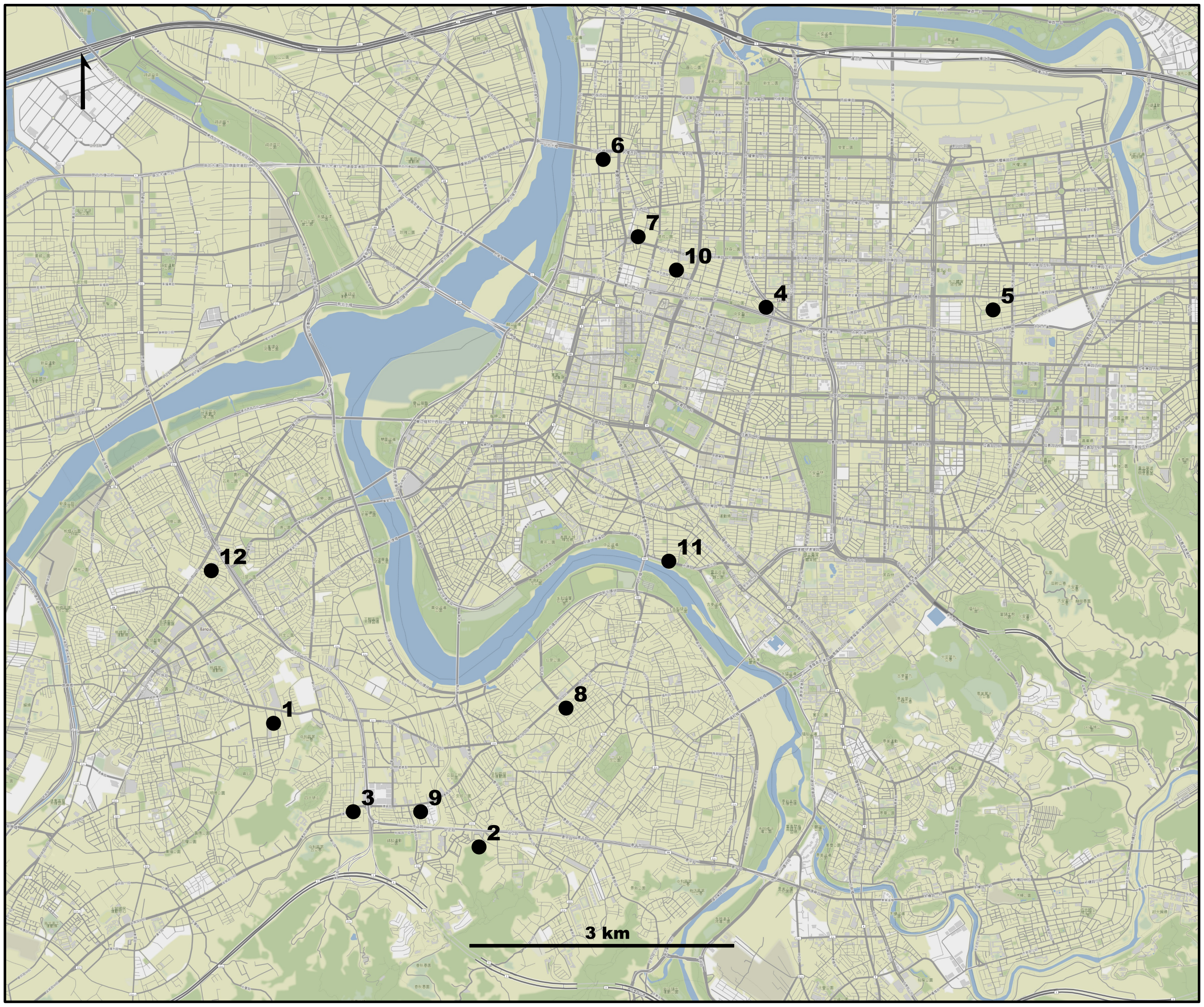

Supplementary Fig. 1 Locations of the 12 residences in Taipei City and New Taipei City in northern Taiwan where sampling was performed. This map was made using QGIS 3.22.4 with Stamen Terrain (by Stamen Design, under CC BY 4.0)

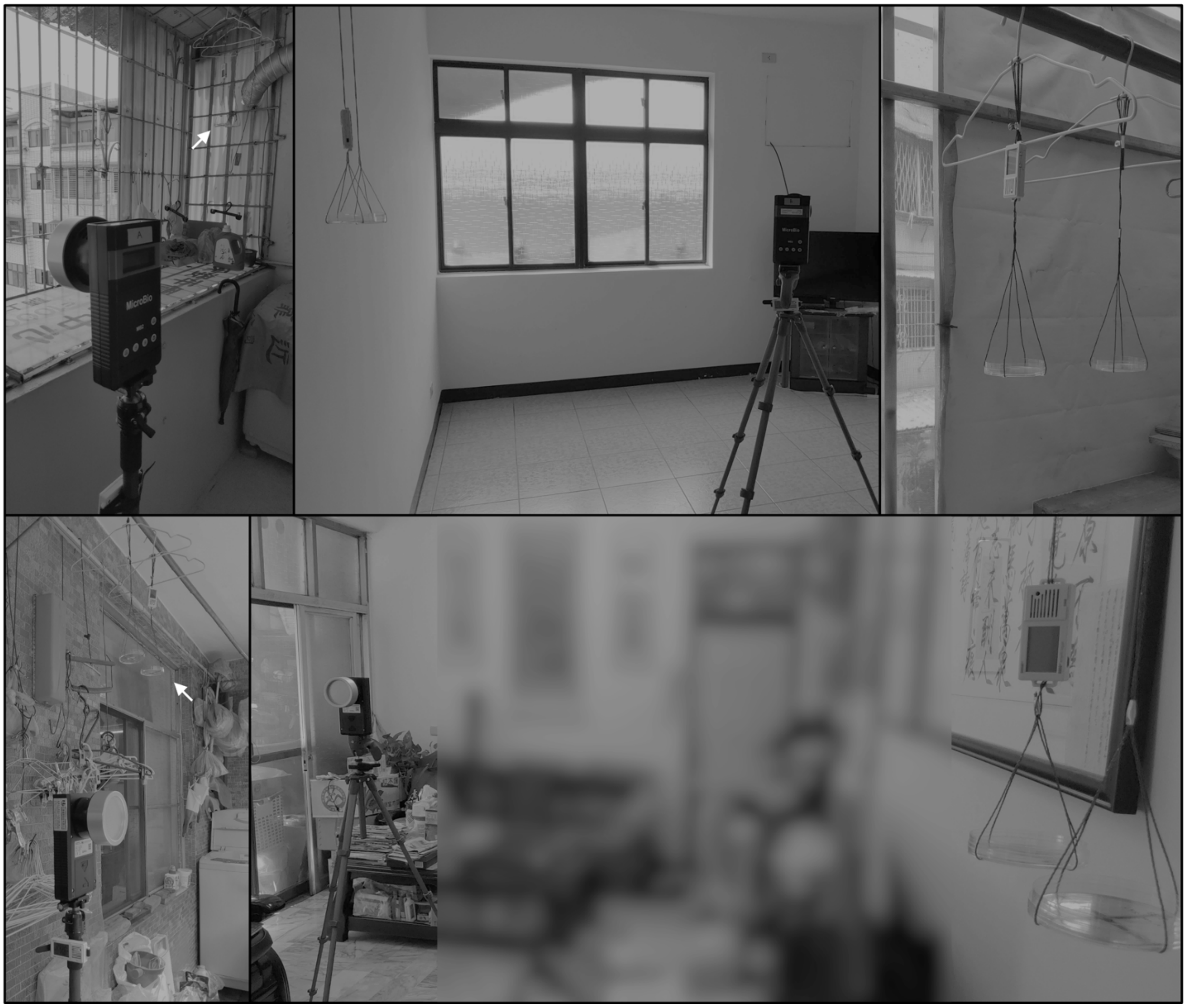

Supplementary Fig. 2 Pictures of indoor and outdoor active and passive air sampling at two residences (top left two and bottom two). The top right picture depicts outdoor passive sampling apparatuses at another residence

Supplementary Table 1 Characteristics of residences and indoor and outdoor mean temperature and relative humidity for 1-day (1 d) and 7-day (7 d) sample periods

Residence no.	Residence type	Residence age (yr)	Floor	No. occupants*	Sample period (yy/mm/dd)	Visit hour (24 h)	Indoor				Outdoor			
							Temperature (°C)		Relative humidity (%)		Temperature (°C)		Relative humidity (%)	
							7 d mean (SD)	1 d mean (SD)	7 d mean (SD)	1 d mean (SD)	7 d mean (SD)	1 d mean (SD)	7 d mean (SD)	1 d mean (SD)
1	Townhouse	40-50	1	2	20/08/18-20/08/25	9	30.6 (0.4)	30.8 (0.4)	68.3 (4.6)	71.1 (4.3)	31.6 (2.9)	32.5 (2.7)	61.0 (13.4)	61.1 (12.5)
2	Apartment	30-40	14	2		11	31.4 (0.6)	31.6 (0.6)	60.4 (5.6)	63.3 (5.8)	31.2 (3.1)	31.6 (3.0)	64.7 (14.3)	66.7 (14.7)
3	Apartment	30-40	5	1		13	31.8 (0.7)	31.8 (0.4)	59.2 (6.9)	60.9 (6.1)	31.6 (2.1)	32.9 (2.2)	61.9 (11.0)	61.1 (11.5)
4	Apartment	40-50	2	3	20/08/21-20/08/28	9	31.2 (1.2)	29.7 (1.2)	63.9 (6.3)	64.9 (5.9)	30.8 (2.6)	28.3 (1.8)	67.5 (12.7)	81.3 (9.1)
5	Apartment	20-30	5	2		11	30.7 (1.2)	30.4 (0.5)	63.3 (7.0)	70.4 (2.4)	31.4 (1.9)	29.7 (1.3)	65.9 (9.9)	76.4 (6.1)
6	Apartment	40-50	5	2		13	30.5 (0.9)	29.6 (0.4)	65.8 (6.0)	72.1 (2.3)	30.7 (2.9)	28.5 (2.6)	68.0 (13.6)	80.3 (12.0)
7	Townhouse	50-60	1	2	20/09/02-20/09/09	9	29.9 (0.8)	30.1 (0.4)	60.5 (8.9)	61.3 (3.1)	29.6 (2.3)	30.0 (1.8)	62.3 (12.5)	62.2 (7.8)
8	Apartment	40-50	6	1		11	31.6 (1.7)	31.4 (1.2)	53.5 (8.4)	57.0 (4.3)	30.6 (2.7)	30.8 (2.4)	56.4 (12.3)	59.6 (8.9)
9	Apartment	40-50	2	2		13	30.7 (0.6)	30.4 (0.2)	58.0 (8.8)	63.7 (3.2)	29.7 (2.0)	29.6 (1.7)	61.3 (11.1)	66.6 (6.2)
10	Apartment	30-40	4	4	20/09/04-20/09/11	9	28.6 (1.8)	28.9 (1.6)	57.1 (7.9)	65.9 (4.5)	29.3 (1.5)	29.0 (1.1)	63.2 (11.0)	74.1 (5.0)
11	Apartment	20-30	9	2		11	29.8 (1.3)	29.6 (1.1)	60.4 (9.8)	69.9 (4.1)	29.5 (1.4)	29.5 (1.2)	61.7 (10.4)	71.2 (4.9)
12	Apartment	50-60	4	3		13	30.9 (0.6)	31.0 (0.4)	54.9 (8.5)	64.8 (3.9)	30.8 (1.5)	30.9 (1.3)	56.9 (9.6)	66.4 (6.5)

*No children, mainly elderly (> 65 years old)

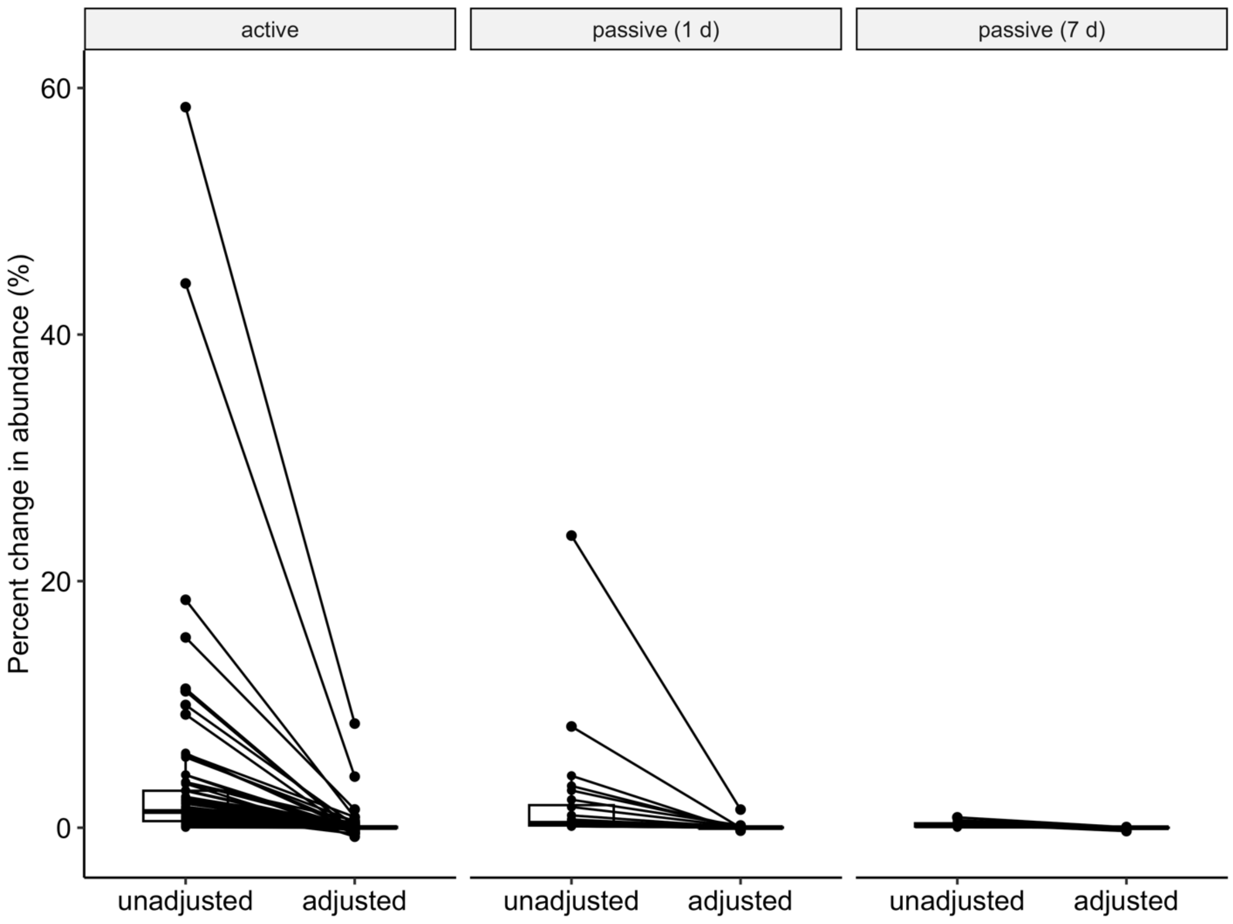

Supplementary Fig. 3 Comparison of percent change in abundance due to rounding after estimation of genus spore equivalents using the original (unadjusted) and adjusted abundance (active, *n* = 72; 1-day passive, *n* = 24; 7-day passive, *n* = 24)

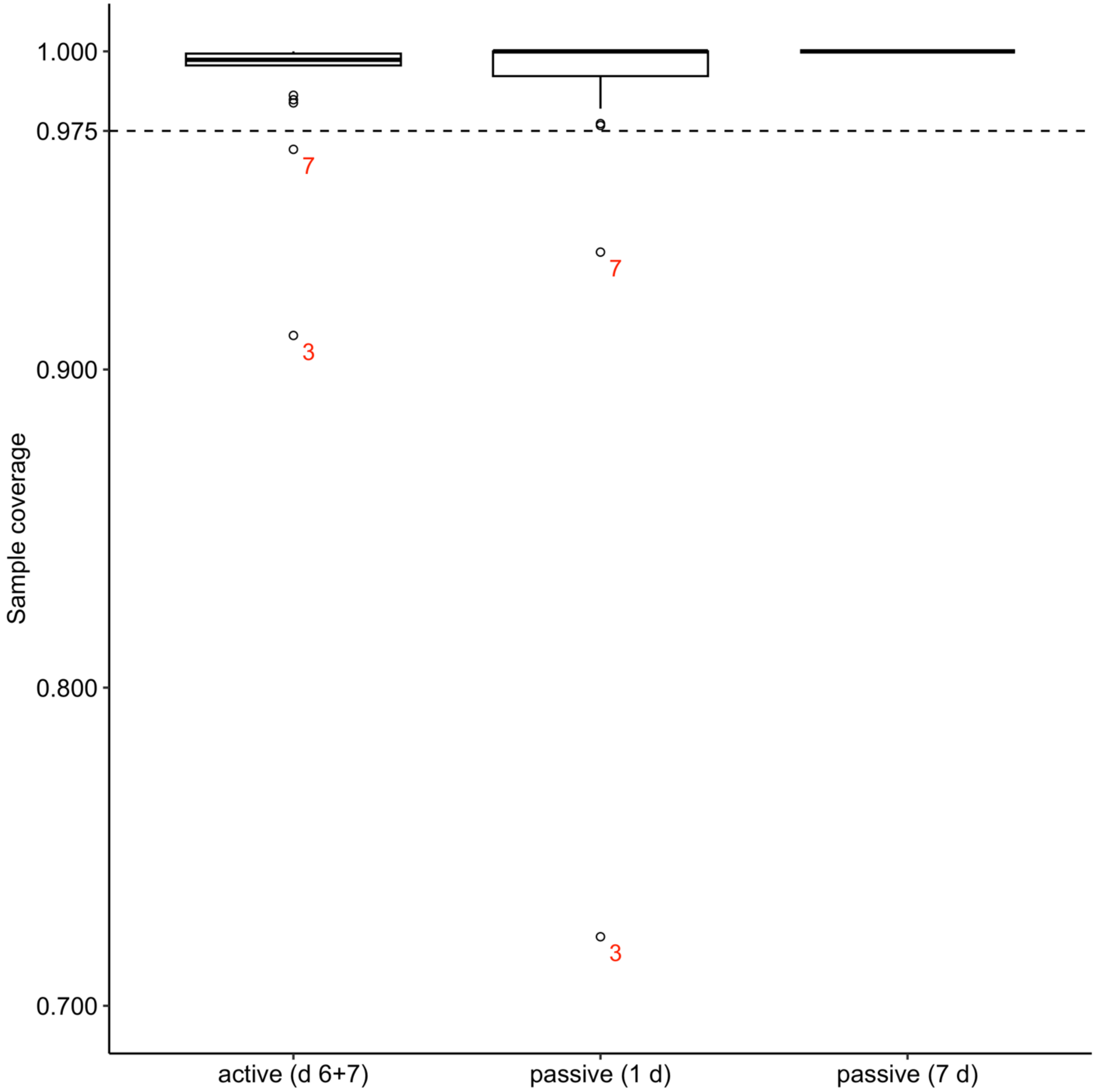

Supplementary Fig. 4 Sample coverage of active and passive air samples (n = 24 per group). Residences no. 3 and no. 7 had samples with coverage < 97.5% (labeled in red)†

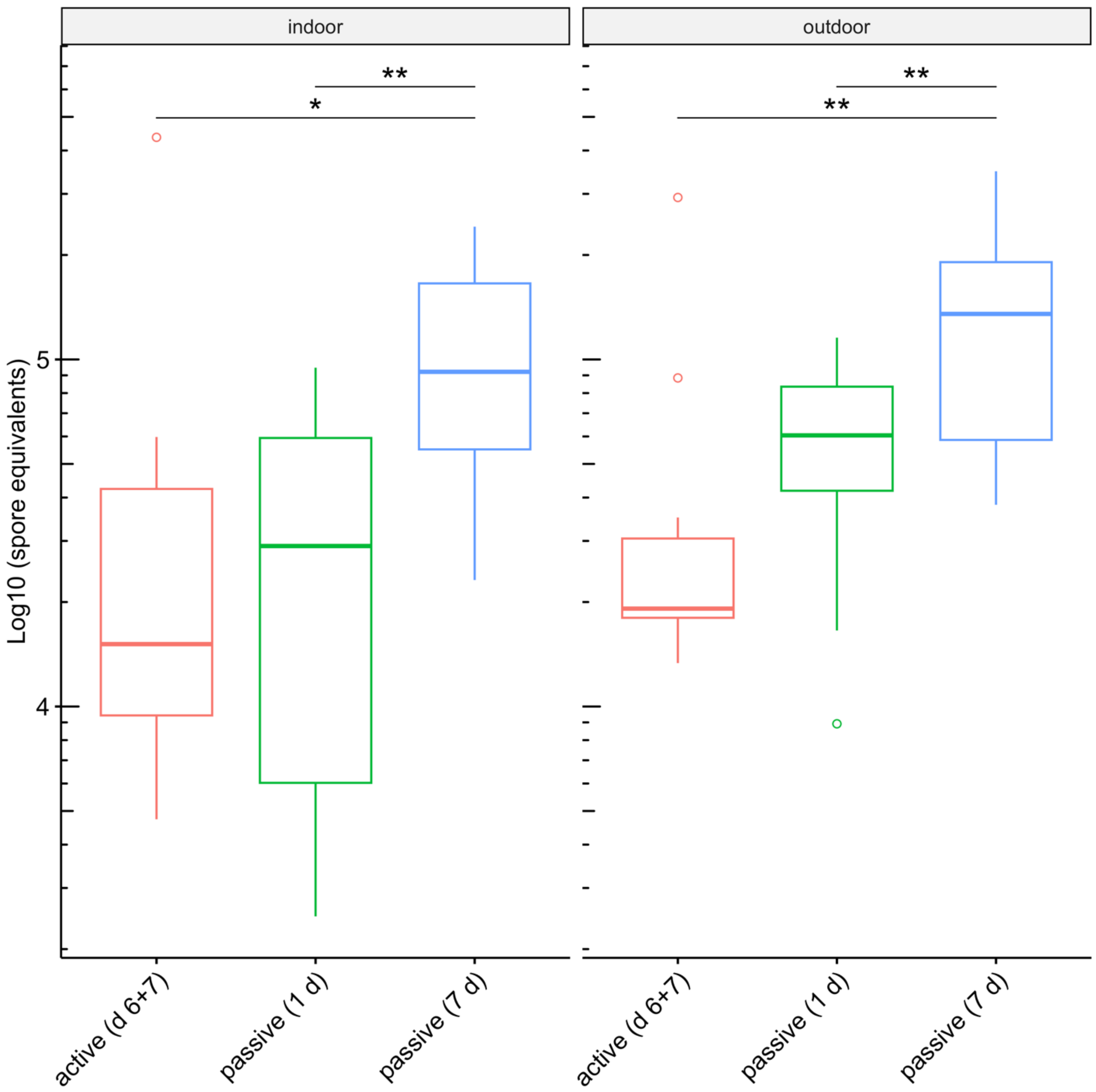

Supplementary Fig. 5 Fungal spore abundance (log10-transformed spore equivalents) between active and passive air samples, stratified by location (*n* = 10 per group)†,‡

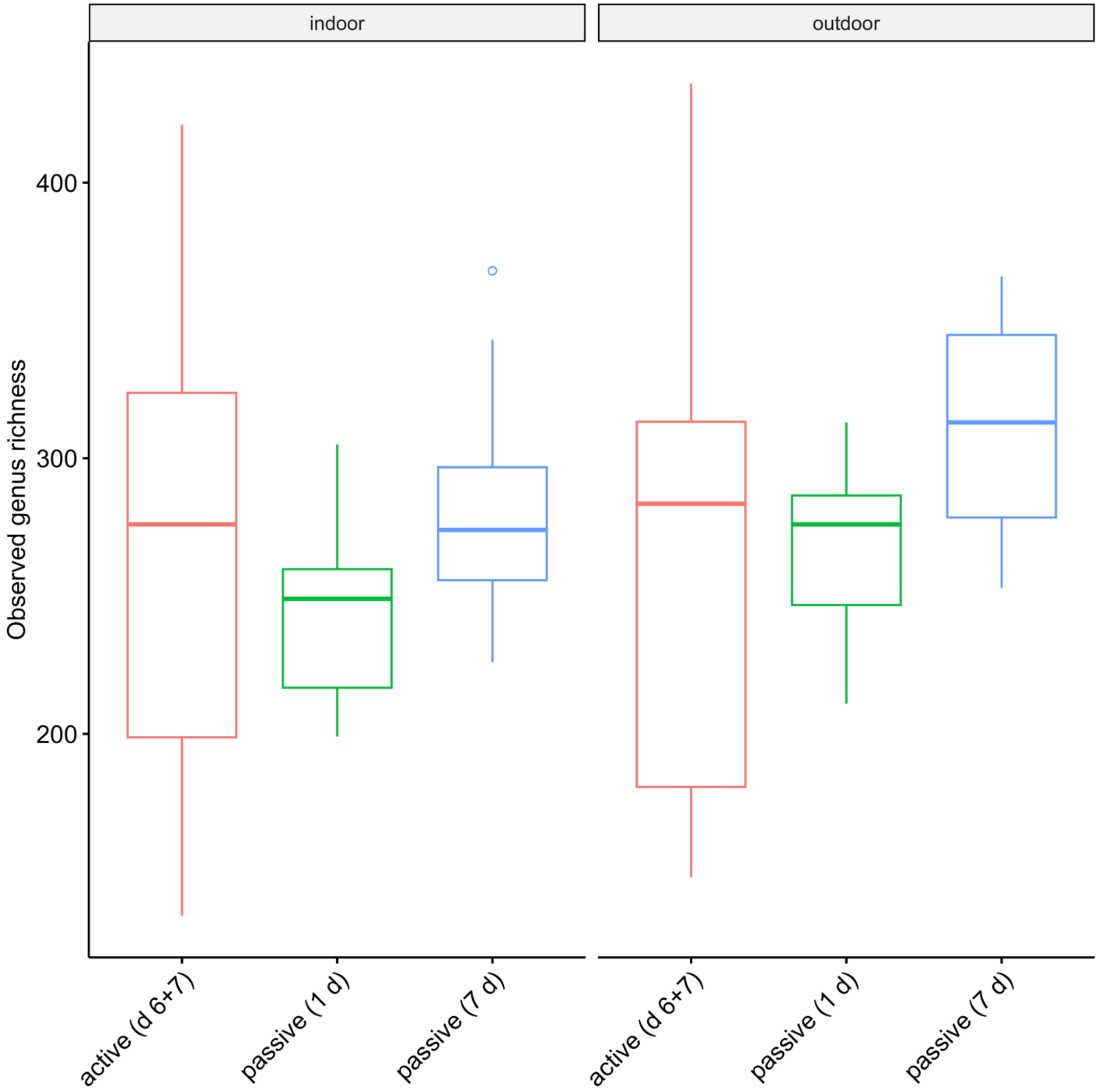

Supplementary Fig. 6 Observed genus richness of fungal spores between active and passive air samples, stratified by location (*n* = 10 per group)[†,§]

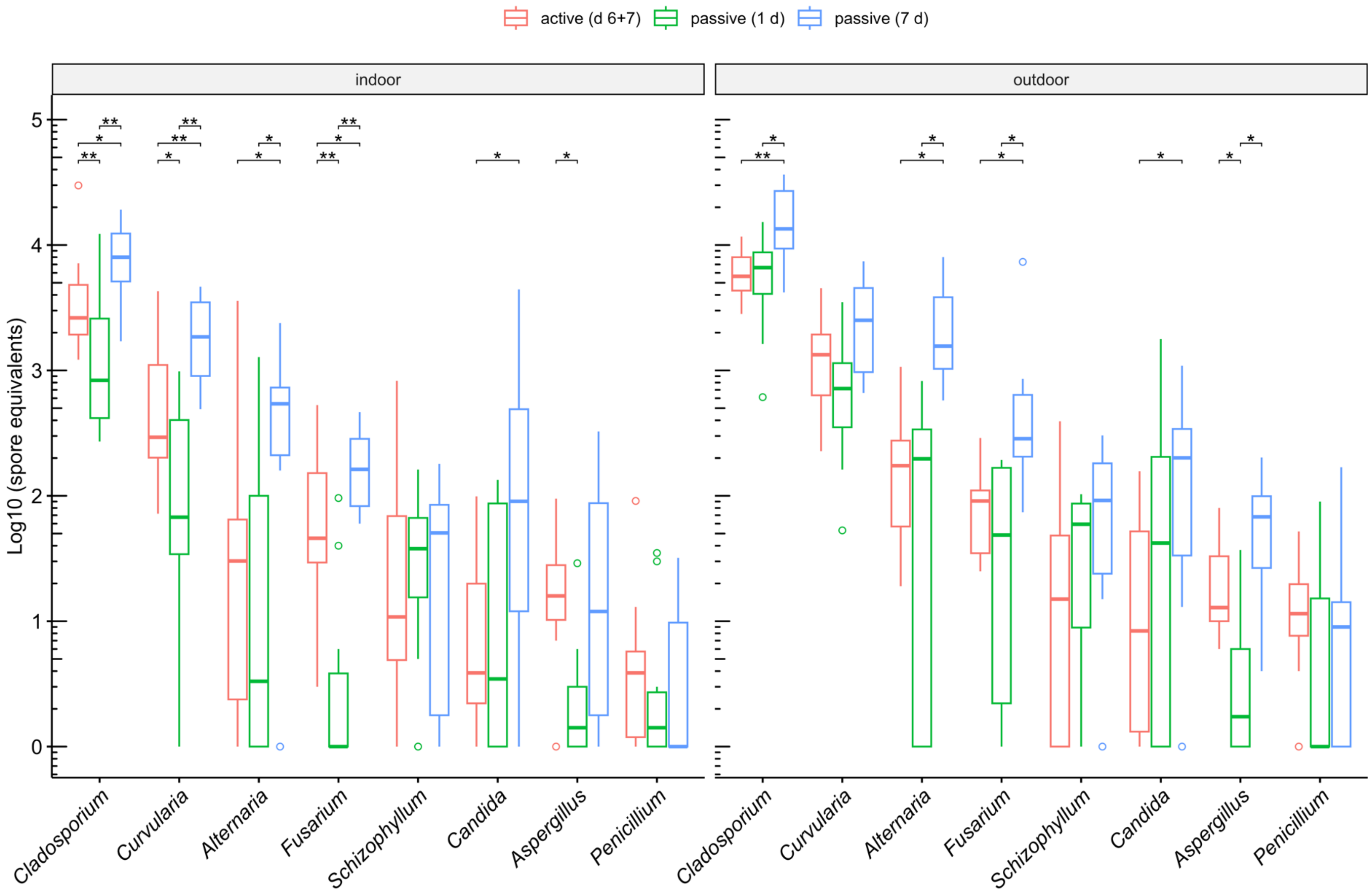

Supplementary Fig. 7 Non-rarefied abundance (log10-transformed spore equivalents) of allergenic genera between active and passive air samples, stratified by location (*n* = 10 per group). Pseudocounts of 1 were added to all values before log10-transformation†,§

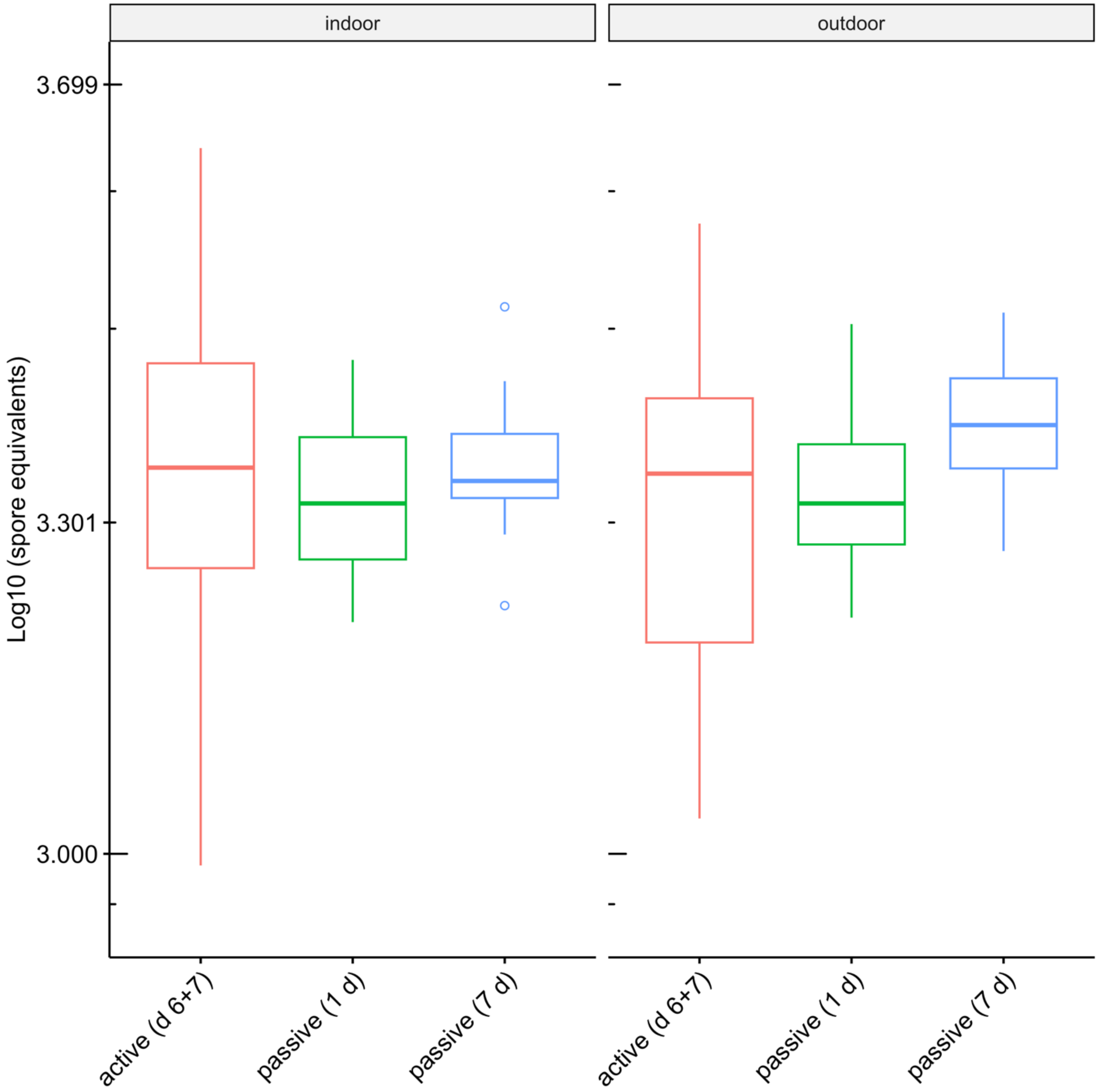

Supplementary Fig. 8 Subsample sizes (*m*) of fungal spore abundance (log10-transformed spore equivalents) used for rarefaction at 97.5% coverage between active and passive air samples, stratified by location (n = 10 per group)[†,§]

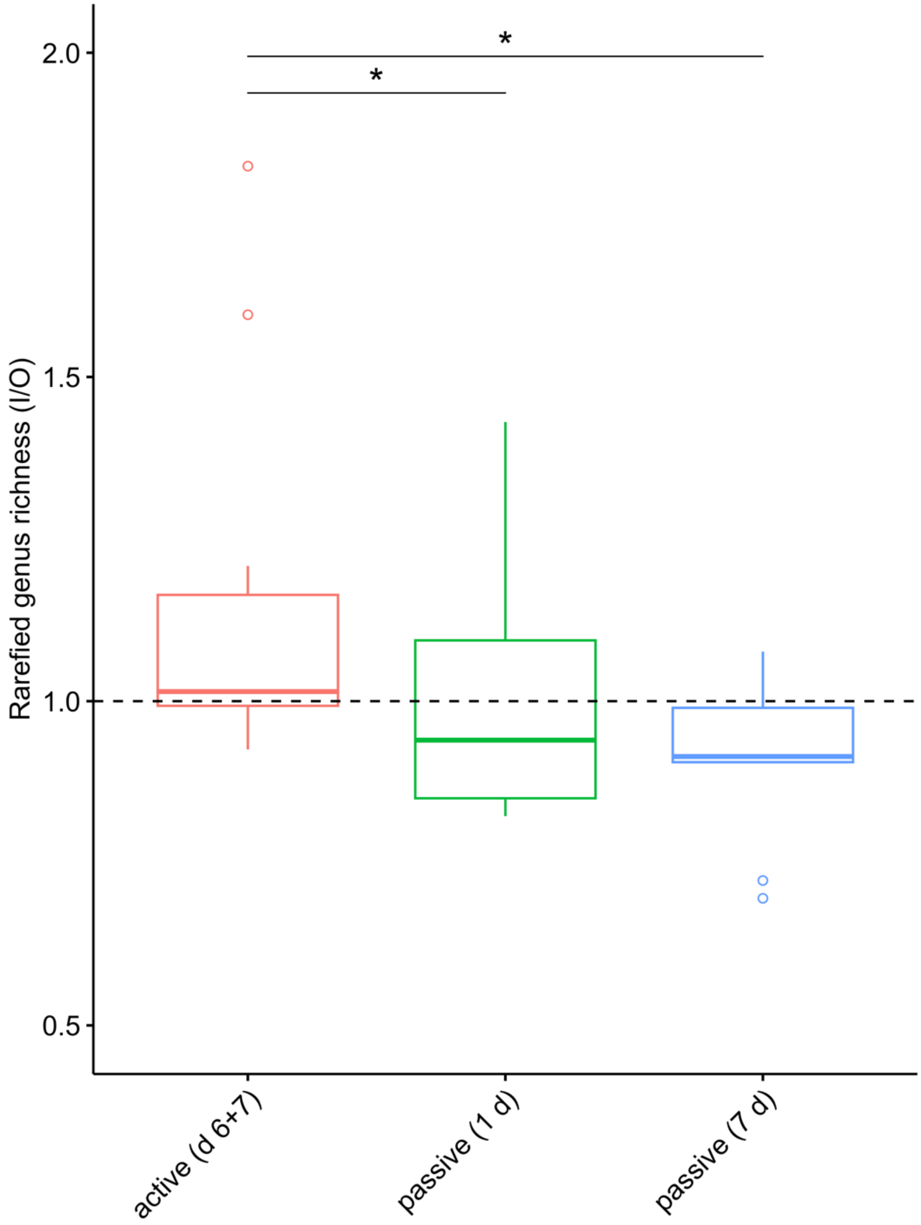

Supplementary Fig. 9 Indoor/outdoor (I/O) ratios of rarefied genus richness of fungal spores between active and passive air samples at a coverage of 97.5% (n = 10 per group)[†,§]

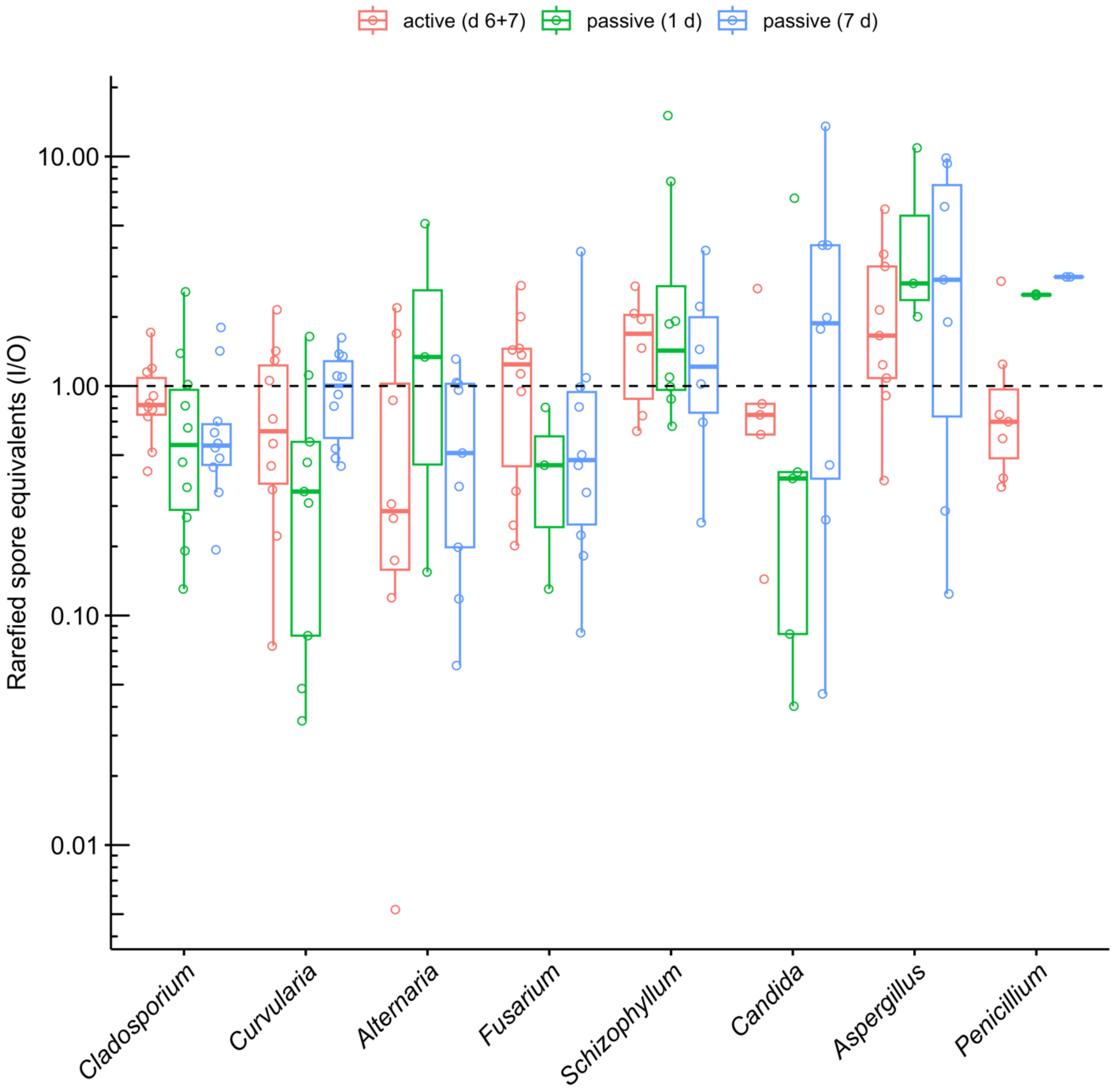

Supplementary Fig. 10 Indoor/outdoor (I/O) ratios of rarefied abundance of allergenic fungal genera between active and passive air samples at a coverage of 97.5%#

†Horizontal lines indicate median, boxes indicate interquartile range (IQR), whiskers indicate values within 1.5 × IQR of Q1 and Q3, and open circles indicate outliers

‡Statistical comparisons were made using paired *t*-test, and *p* values were adjusted for multiple comparisons using the Holm method ($p \geq 0.05$ not shown; * = $p < 0.05$; ** = $p < 0.01$)

§Statistical comparisons were made using Wilcoxon signed-rank exact test, and *p* values were adjusted for multiple comparisons using the Holm method ($p \geq 0.05$ not shown; * = $p < 0.05$; ** = $p < 0.01$)

#Horizontal lines indicate median, boxes indicate IQR, whiskers indicate values within 1.5 × IQR of Q1 and Q3, and all data points are shown as open circles to indicate sample sizes with points plotted beyond the whiskers representing outliers; statistical comparisons were made using Mann–Whitney *U* test, and *p* values were adjusted for multiple comparisons using the Holm method ($p \geq 0.05$ not shown)
